# TOWARD SUCCESSFUL IMPLEMENTATION OF A QUALITY IMPROVEMENT INTERVENTION IN NURSING HOMES: EXTERNAL FACILITATION

**DOI:** 10.1093/geroni/igae098.3418

**Published:** 2024-12-31

**Authors:** Regina George, Jacob Miranda, A Lynn Snow, Michelle Hilgeman, Megan McCullough, Christine Hartmann

**Affiliations:** University of Alabama, Tuscaloosa, Alabama, United States; California State University East Bay, Hayward, California, United States; University of Alabama, Tuscaloosa, Alabama, United States; Tuscaloosa VA Medical Center, Tuscaloosa, Alabama, United States; University of Massachusetts Lowell, Lowell, Massachusetts, United States; VA Bedford Healthcare System & University of Massachusetts Lowell, Bedford, Massachusetts, United States

## Abstract

Nursing home interventions are challenging to implement and sustain. Implementation scientists have identified facilitation as a strategy that impacts the success of an intervention. Facilitation is a complex and dynamic process that requires facilitators to develop expertise in a wide range of activities necessary to effectively support the implementation. There is limited understanding of how these activities are executed in a dynamic facilitation process. This understanding gap impacts training of facilitators and consequently, efficient delivery and sustainment of interventions. A qualitative analysis of the real-time interactions between implementation facilitators and recipient sites would contribute to our understanding of the practice of facilitation in nursing homes. In this study an expert external facilitator’s interactions with nursing home teams were recorded and the activities of the external facilitator (EF) were analyzed. Data comes from a pilot three-site pragmatic clinical trial of a complex quality improvement and sleep intervention for residents living with dementia in nursing homes. The intervention is based in relational coordination, person-centered care, and adult learning principles. EF largely took place in leadership team 30-minute coaching sessions via videoconference. Reflexive thematic analysis of 24 EF session transcripts is ongoing: preliminary code clusters include metacognition (connecting actions and impact), strategic compliments (praise that encourages specific action) and problem solving (connecting causes and developing individualized solutions). Transcripts provide real-world examples of EF activities. This project provides insight into the practice of facilitation and may inform training of implementation facilitators, thus increasing incorporation and sustainment of interventions in nursing homes.

